# The impact of work related physical activity and leisure physical activity on the risk and prognosis of neck pain – a population based cohort study on workers

**DOI:** 10.1186/s12891-016-1080-1

**Published:** 2016-05-20

**Authors:** Lina Palmlöf, Lena W. Holm, Lars Alfredsson, Cecilia Magnusson, Eva Vingård, Eva Skillgate

**Affiliations:** The Institute of Environmental Medicine, Musculoskeletal & Sports Injury Epidemiology Center, Karolinska Institutet, Box 210, 171 77 Stockholm, Sweden; The Institute of Environmental Medicine, Karolinska Institutet, Box 210, 171 77 Stockholm, Sweden; The Institution of Public Health Sciences, Tomtebodavägen 18a, Widerströmska Huset, 171 77 Stockholm, Sweden; Department of Medical Science, Occupational and Environmental Medicine, Akademiska sjukhuset, Ulleråkersvägen 40, 751 85 Uppsala, Sweden; Naprapathögskolan - Scandinavian College of Naprapathic Manual Medicine, Kräftriket 23A, 11419 Stockholm, Sweden

**Keywords:** Epidemiology, Neck pain, Prognosis, Risk factor, Physical activity, Cohort study, Longitudinal study

## Abstract

**Background:**

The effect of physical activity on risk and prognosis for neck pain has been studied earlier with inconclusive results. There is a need for large prospective studies on the subject. The aim of this study was to investigate if work related physical activity and physical activity during leisure time are of importance for the risk and prognosis of neck pain in men and women.

**Methods:**

We used the Stockholm Public Health Cohort and formed two sub-cohorts of the working population based on data from 2002. Cohort I (risk cohort) included persons without neck pain (*n* = 4681), and cohort II (prognostic cohort) included persons with occasional neck pain (*n* = 6820) during the previous six months. Both cohorts were assessed for the outcome long duration troublesome neck pain (LDNP) in 2007.

The exposures and potential confounders were assessed through a questionnaire in 2002. The question regarding work related physical activity over the past 12 months had five answering categories ranging from “sedentary” to”heavy”. The question regarding leisure physical activity for the past 12 months had five answering categories ranging from “sedentary” to “regular physical activity”.

LDNP in 2007 was defined as having had troublesome neck pain lasting ≥ 3 consecutive months during the previous five years. Associations between work related physical activity and LDNP, as well as leisure physical activity and LDNP, were investigated by multivariable logistic regression, considering potential confounding factors.

**Results:**

In cohort I (risk cohort) we found an association between leisure physical activity and LDNP. In cohort II (prognostic cohort) we found no association between the exposures and the outcome.

**Conclusion:**

The results suggest that leisure physical activity has a protective effect on the risk of developing LDNP in a population free from neck pain. It did not, however, affect the prognosis of occasional neck pain. Neither the risk nor the prognosis of neck pain was affected by work related physical activity in this study.

## Background

Physical activity has a positive influence on health according to several studies. Being physically active can decrease the risk of onset of disease (e.g. coronary heart disease and depression) [1] as well as act as a treatment for already established disease (e.g. rheumatoid arthritis) [2]. Today sedentary behavior is common both at the workplace and during leisure time. It is known that being sedentary for a long period of time during the day affects one’s health negatively, even for those who meet the public health recommendations for weekly physical activity [3].

Neck pain is a common disorder in the general population with a 12-month prevalence between 30–50 % [4] and for bothersome neck pain the 12-month prevalence is 16 % among men and 25 % among women [5]. Of those who recover from neck pain, up to 75 % will relapse within 1–5 years [6]. The societal cost for neck pain is burdensome, due to, for example, sick-leave expenses [7]. Considering its recurring nature and that neck pain is a big societal as well as individual burden, it is of great importance to identify what factors that may influence the risk and prognosis of neck pain.

Levels of leisure physical activity has previously been studied as a potential risk factor for onset of neck pain in several studies with inconsistent results [4]. However, a low leisure physical activity has been seen to be associated with other pain related disabilities such as low back pain and multisite musculoskeletal pain [8, 9]. When investigated as a prognostic factor for neck pain, the authors of a review study suggested that a higher level of physical activity indicates a more favorable prognosis, [10] although they concluded the results to be preliminary [10]. The remaining uncertainty whether physical activity is of importance for the risk for and/or prognosis of neck pain underpins a need for large population based studies on this matter.

The increased blood flow [11] and analgesic effect, [12] as a result of physical activity, could potentially affect both the risk for, and the prognosis of, neck pain. As physical activity is a modifiable factor, it is highly interesting to investigate its association with neck pain. If such associations were to be present, it would be possible to affect the risk for, as well as the course of, neck pain through lifestyle changes, and consequently decrease individual suffering and societal costs.

Physical activity can be carried out in different arenas and settings. Therefore, to get a more complete picture of the effects of physical activity on neck pain we wanted to investigate both physical activity performed during leisure time, as well as work related physical activity. We hypothesize that being physically active is protective of neck pain, regardless of whether the activity is performed within the context of a work or during leisure time. We have access to a large population based study with extensive baseline measurements and longitudinal information on neck pain. Thus, the aim of our study was to investigate if work related physical activity and physical activity during leisure time are of importance for the risk and prognosis of neck pain. Due to a demand for sex-specific studies on this matter, [13] we chose to investigate each association in the present study for men and women collectively as well as separately.

## Methods

### Source population and data collection

This cohort study was based on the Stockholm Public Health Cohort (SPHC), which is a population based, longitudinal study [14]. The segment of SPHC used in this study had its baseline conducted in 2002 and a follow-up in 2007 (*n* = 23 794). The source population constitutes of residents in the ages of 18–65 years, from 24 out of 26 municipalities in Stockholm County, Sweden. This geographical area is an urban environment with a total of 1.4 million inhabitants. From this source population, the study sample of 50 067 persons was randomly selected after stratification for gender and residential area. Between October 2002 and March 2003, these persons received a baseline questionnaire, which was followed by a maximum of three reminders in absence of reply. Participants gave their informed consent to take part in the study by answering the baseline questionnaire. The baseline questionnaire was returned by 62 % (31 182 persons), and the responders were sent a follow-up questionnaire between March and August 2007. The questionnaires were extensive and contained questions on, for example, demographic characteristics, life style factors, physical and mental health. The study was approved by the Stockholm Regional Ethics Committee, ref. no.2009/5:4.

### Study populations

In order to investigate the working population we excluded those who were neither employed nor self-employed, as well as those over 60 years of age at baseline. Two sub cohorts were formed, one for analyses on risk and one for analyses on prognosis of neck pain. *Cohort I* was based on individuals who reported no neck pain during the past 6 months in the baseline questionnaire (*n* = 4 681), and these individuals were assessed for the risk of onset of long duration troublesome neck pain.

**Fig. 1:**
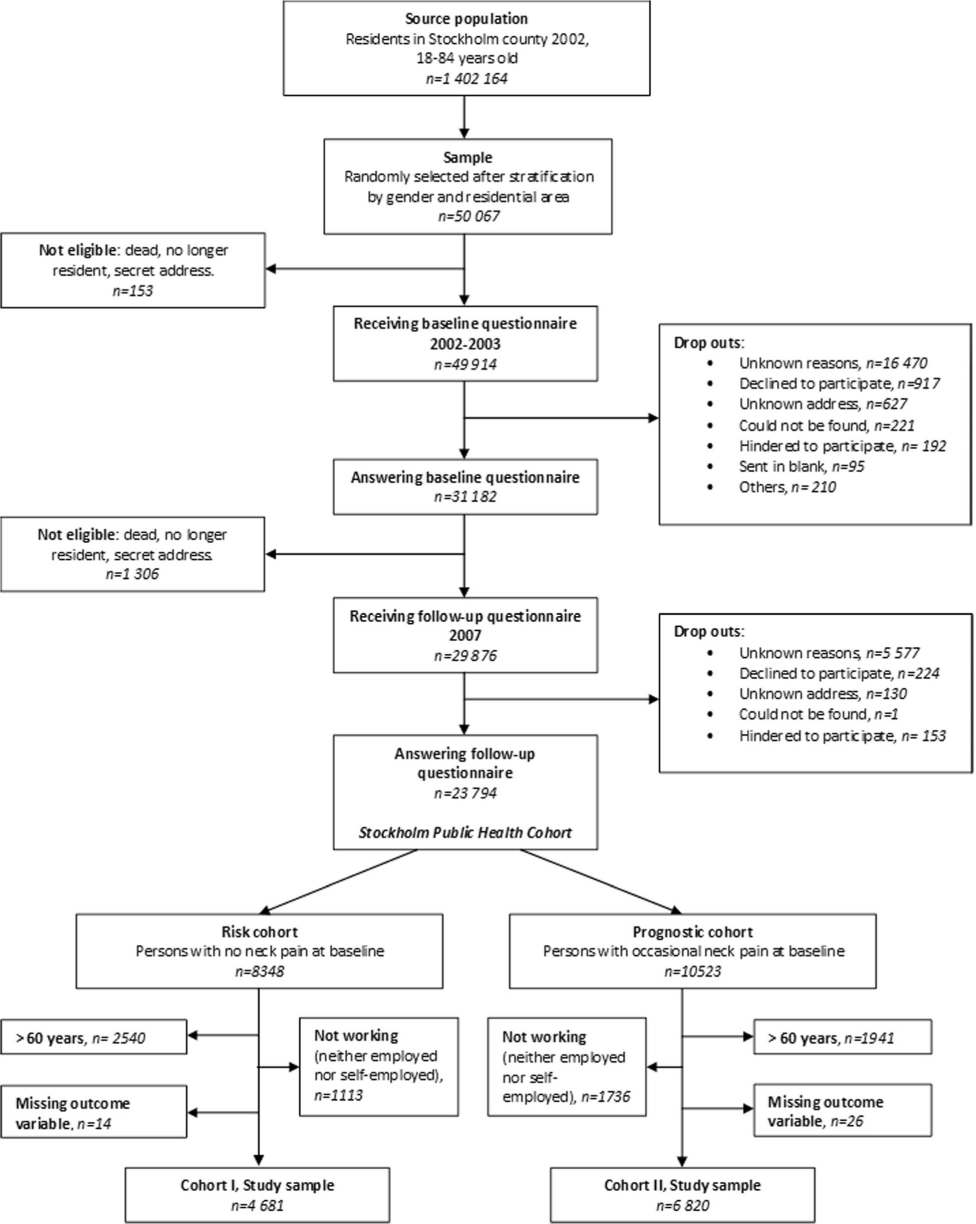
Flowchart of inclusion process for the study

Studying prognosis requires a study population with the disease under study at baseline. Thus, for the assessment of prognosis of neck pain, *cohort II* included individuals with occasional neck pain at baseline, defined as having had neck pain up to a couple of days per month during the previous six months according to the baseline questionnaire (*n* = 6 820). These individuals were assessed for the same outcome as individuals in cohort I; risk of onset of long duration troublesome neck pain. Flow chart of participant recruitment is shown in Fig. 1.

### Exposures

Work related physical activity was assessed in the baseline questionnaire with the question; “How much have you been physically active or exerted yourself physically at your work during the past twelve months?” The answering categories were; 1) Sedentary work: you have a predominantly sedentary work, 2) Light but somewhat active work: you have a work situation where you walk quite a lot but do not carry or lift heavy things, 3) Moderately heavy work: you walk a lot as well as lifting quite a lot and also climb stairs or walk uphill, and 4) Heavy work: you have a heavy manual labor, lifting heavy things and physically exert yourself to a high degree. The question is considered to have good validity and reproducibility [15].

Leisure physical activity was also subjectively reported in the baseline questionnaire through the following question; “How much have you been physically active in your leisure time during the past twelve months?” The participants were asked to state an average if their leisure time physical activity varied over the seasons. The four answering categories were; 1) Sedentary leisure time: you spent most of your time reading, watching TV, going to the movies or other sedentary activity during leisure time. You walk, ride a bike or engage in physically active in some other way less than 2 h/week, 2) Moderate physical activity during leisure time: You walk, ride a bike or engage in physical activity in some other way for a minimum of 2 h/week, often without perspiring. This also includes walking or riding a bike to or from work, Sunday walks, ordinary gardening, fishing, table tennis and bowling, 3) Moderate, regular physical activity during leisure time: you exercise regularly 1–2 times/week, each time for a minimum of 30 min, with jogging, swimming, tennis, badminton or other activity that makes you perspire, and 4) Regular physical activity and exercise: you are active with running, swimming, tennis, badminton, aerobics or something similar at least three times/week. Each occasion lasts for at least 30 min. The leisure physical activity question has acceptable validity when compared to results from an accelerometer [16].

In the analyses of the association between work related physical activity and physical activity during leisure time and the risk of LDNP in the risk cohort (cohort I) was the two highest categories of physical activity merged due to lack of statistical power.

### Outcome

The outcome in the analyses of both *cohort I* and *cohort II* was long duration troublesome neck pain (LDNP). Those who classified as cases were those who answered “yes” to the following question in the follow-up questionnaire; “During the last five-year period, have you had neck pain for at least three consecutive months that bothered you considerably?” This definition is in line with the conceptual framework for the definition of neck pain outlined by the Neck pain task force [17].

### Potential confounders

Information from the baseline questionnaire was used to identify potential confounders in the analyses of associations between the exposures work related physical activity and leisure physical activity and the outcome LDNP. They were selected with guidance from previous studies and literature on risk and prognostic factors for neck pain. The following variables were evaluated as potential confounders; immigrant status (born in or outside of Sweden), alcohol consumption (expressed in gram of 100 % alcohol per day and categorized into no, low, moderate and high level of consumption), smoking (categorized into never, ever and current smoker), concurrent low back pain (during the past six months measured on a five level scale ranging from “never” to “daily”), and individual disposable income (individualized share of family income based on income, social benefits and tax deductions, categorized into quartiles). The baseline questionnaire contained some of the questions from the Job Content Questionnaire (JCQ), concerning psychosocial occupational work load [18]. Of the available questions we chose to test two as potential confounders; “freedom to decide how the work shall be performed”, and “freedom to decide what should be performed” (both with four answering categories ranging from “never” to “always”). Also considered as potential confounders were “Working with arms above shoulder level at least 30 min per day” and “working with arms under knee level at least 30 min per day” (both with five answering categories ranging from “Almost never or never” to “Every day”). The portion of the workday spent in front of a computer (with six answering categories ranging from “Never” to “Almost all the time”) was tested as potential confounder only in the analyses of associations between leisure physical activity and LDNP since this factor is highly correlated with sedentary work. Furthermore we evaluated amount of time that work is considered to be stressful (categorized into five, ranging from “about 1/10 of the working hours” to “almost all working hours”) and also levels of psychological distress (measured by the 12-item General Health Questionnaire (GHQ12)) with regard to potential confounding [19]. A sum score of ≥ 3 (using the recommended standard 0-0-1-1 scoring on the four answering alternatives) was used to denote psychological distress. Sex was tested as a confounder in the analysis of both sexes. Lastly we evaluated work related physical activity as a potential confounder for the associations between leisure physical activity and the outcome, and vice versa. Age was divided into five categories (18–25, 26–35, 36–45, 46–55 and >55) because of its non-linear relationship with LDNP and was included as a covariate in all final models.

### Statistical analysis

Associations between the exposures and the outcome were assessed by multivariable logistic regression and the results were presented as odds ratios (OR) with 95 % confidence intervals (95 % CI). Multivariable logistic regression models were built for each exposure and sex, in *cohort I* and *cohort II* respectively, and also one including both sexes for each exposure and cohort. All potential confounders were entered one at a time in each of the models. If the crude odds ratio was changed with more than 10 %, the variable was considered to be a confounder for that specific association and was included as a covariate in the final model [20]. Individuals with missing data on the outcome or exposure were excluded from the analyses. Statistical analyses were run with STATA® statistical software system V.11.

## Results

### Cohort I - Risk cohort

The baseline characteristics of the study population (*n* = 4 681) are shown in Table 1. The participants had a mean age of 44 (SD 11) and 60 % were men.

**Table 1:** Participants’ baseline characteristics

	Cohort I (risk cohort), individuals with no neck pain at baseline (*n* = 4 681)		Cohort II (prognostic cohort), individuals with occasional neck pain at baseline (*n* = 6 820)	
	Cases n (%)	Non cases n (%)	Cases n (%)	Non cases n (%)
N	177	4 504	766	6 054
Male sex	93 (53)	2 701 (60)	299 (39)	2 584 (43)
Age, mean (SD)	42.82 (9.90)	43.80 (10.95)	42.26 (9.89)	41.85 (10.87)
Individual disposable income (SEK^a^)				
0–115 299	48 (27)	745 (17)	214 (28)	1 253 (21)
115 30–154 399	44 (25)	988 (22)	172 (23)	1 417 (24)
154 200 – 207 499	43 (25)	1 227 (27)	199 (26)	1 658 (28)
≥ 207 500	40 (23)	1 519 (34)	172 (23)	1 686 (28)
Swedish born				
No	42 (24)	472 (11)	199 (26)	823 (14)
Yes	135 (76)	4 023 (89)	565 (74)	5 211 (86)
Smoking (%)				
Never	92 (54)	2 146 (50)	328 (45)	2 709 (47)
Ever	28 (16)	916 (21)	152 (21)	1 174 (21)
Current	50 (29)	1 212 (28)	247 (34)	1 839 (32)
SEI ^b^				
Unskilled manual workers	29 (17)	598 (14)	141 (19)	915 (15)
Skilled manual workers	17 (10)	318 (7)	94 (13)	584 (10)
Lower non-manual workers	19 (11)	532 (12)	101 (14)	843 (14)
Intermediate non-manual workers	47 (27)	1 196 (27)	193 (26)	1 690 (28)
Higher non-manual workers	38 (22)	1 298 (29)	138 (19)	1 419 (24)
Self-employed	22 (13)	489 (11)	79 (11)	515 (9)
Psychological distress (12-item General Health Questionnaire)				
No	135 (79)	3 854 (87)	538 (71)	4 671 (78)
Yes	35 (21)	569 (13)	221 (29)	1 345 (22)
Alcohol consumption (Expressed in grams of 100 % alcohol per day)				
No	24 (14)	368 (8)	91 (12)	526 (9)
Low	128 (75)	3 519 (79)	549 (74)	4 578 (77)
Moderate	13 (8)	435 (10)	87 (12)	697 (12)
High	5 (3)	113 (3)	15 (2)	130 (2)
Concurrent low back pain				
No, never	91 (51)	2 677 (60)	164 (22)	1 564 (26)
Yes, a couple of days the past 12 months	44 (25)	1 191 (27)	311 (41)	2 732 (45)
Yes, a couple of days per month	22 (12)	360 (8)	211 (28)	1 324 (22)
Yes, a couple of days per week	6 (3)	151 (3)	43 (6)	274 (5)
Yes, daily	14 (8)	114 (3)	33 (4)	151 (3)
Do you have the freedom to decide how your work will be performed?				
Never	9 (5)	146 (3)	50 (7)	268 (4)
Mostly not	25 (14)	569 (13)	139 (18)	1 105 (18)
Yes, most of the time	108 (62)	2 749 (62)	435 (58)	3 727 (62)
Always	32 (18)	997 (22)	126 (17)	903 (15)
Do you have the freedom to decide what to perform at your work?				
Never	18 (10)	302 (7)	87 (12)	541 (9)
Mostly not	55 (32)	1 253 (28)	241 (32)	2 022 (34)
Yes, most of the time	77 (44)	2 360 (53)	341 (45)	2 954 (49)
Always	24 (14)	546 (12)	84 (11)	482 (8)
Do you have a stressful job?				
Somewhat, about 10 % of the time	63 (36)	1 854 (42)	279 (37)	2 068 (35)
Yes, about 1/4 of the time	29 (17)	774 (18)	117 (16)	1 134 (19)
Yes, half the time	27 (16)	656 (15)	134 (18)	1 018 (17)
Yes, about 3/4 of the time	31 (18)	579 (13)	126 (17)	973 (16)
Yes, almost the whole time	24 (14)	544 (12)	92 (12)	768 (13)
Do you work with your arms above shoulder level more than 30 min/day?				
Almost never or never	128 (78)	3727 (85)	530 (71)	4673 (78)
Yes, 1–3 days per month	5 (3)	166 (4)	40 (5)	290 (5)
Yes, 1 day per week	2 (1)	102 (2)	25 (3)	178 (3)
Yes, 2–4 days per week	6 (4)	119 (3)	48 (6)	256 (4)
Yes, every day	23 (14)	280 (6)	99 (13)	562 (9)
Do you work with your arms below knee level more than 30 min/day?				
Almost never or never	136 (82)	3784 (86)	566 (76)	4831 (81)
Yes, 1–3 days per month	6 (4)	154 (4)	36 (5)	274 (5)
Yes, 1 day per week	4 (2)	77 (2)	32 (4)	141 (2)
Yes, 2–4 days per week	7 (4)	118 (3)	37 (5)	229 (4)
Yes, every day	12 (7)	260 (6)	71 (10)	487 (8)
How big part of the day do you work in front of the computer?				
Not at all	33 (20)	751 (17)	186 (25)	1205 (20)
Maximum 1/10	33 (20)	825 (19)	123 (17)	1053 (18)
About 1/4	28 (17)	718 (16)	91 (12)	813 (14)
About 1/2	29 (18)	659 (15)	93 (13)	842 (14)
About 3/4	11 (7)	635 (14)	96 (13)	828 (14)
Almost the whole day	28 (17)	795 (18)	154 (21)	1217 (20)

Presented per cohort among cases/non cases of long duration troublesome neck pain at follow-up

^a^Swedish kroner

^b^Socio-economic classification (Occupational class)

The numbers may not add up to the total because of internal missing

#### Work related physical activity

Table 2 displays the crude and adjusted ORs of association between work related physical activity and LDNP among participants with no neck pain at baseline.

**Table 2:** Association between work related physical activity and long duration troublesome neck pain in cohort I^a^

	Both sexes OR (95 % CI)			Men OR (95 % CI)			Women OR (95 % CI)		
Work related physical activity	Cases/non-cases	Crude	Adjusted^b^	Cases/non-cases	Crude	Adjusted^c^	Cases/non-cases	Crude	Adjusted^d^
Sedentary (reference category)	73/2080	1	1	40/1292	1	1	33/788	1	1
Active but not heavy	47/1440	0.9 (0.6–1.3)	0.9 (0.6–1.4)	25/813	1.0 (0.6–1.6)	0.9 (0.5–1.6)	22/627	0.8 (0.5–1.5)	0.7 (0.4–1.3)
Active and moderately heavy or heavy	52/943	1.6 (1.1–2.3)	1.0 (0.6–1.6)	25/578	1.4 (0.8–2.3)	0.6 (0.3–1.4)	27/365	1.8 (1.0–3.0)	1.3 (0.7–2.5)

Presented with odds ratios (ORs) and 95 % confidence intervals (95 % CI)

^a^Cohort I: Risk cohort including individuals with no neck pain at baseline

^b^Adjusted for age, alcohol consumption, individual disposable income, work above shoulder level

^c^Adjusted for age, leisure physical activity, alcohol consumption, individual disposable income and work above shoulder level

^d^Adjusted for age, alcohol consumption, smoking, concurrent low back pain, individual disposable income, psychological distress and work above shoulder level

All adjusted ORs for the two categories of work related physical activity were close to one in the analysis of both sexes combined. There were no clear associations in the adjusted results in the sex-stratified analysis displayed in Table 2.

#### Leisure physical activity

Table 3 displays the crude and adjusted ORs of the associations between leisure physical activity and LDNP among participants with no neck pain.

**Table 3:** Association between leisure physical activity and long duration troublesome neck pain in cohort I^a^

	Both sexes OR (95 % CI)			Men OR (95 % CI)			Women OR (95 % CI)		
Leisure physical activity	Cases/non- cases	Crude	Adjusted^b^	Cases/non-cases	Crude	Adjusted^c^	Cases/non-cases	Crude	Adjusted^d^
Sedentary (reference category)	37/534	1	1	19/341	1	1	18/193	1	1
Moderate	66/1802	0.5 (0.3–0.8)	0.7 (0.4–1.0)	37/1024	0.6 (0.4–1.1)	0.8 (0.4–1.6)	29/778	0.4 (0.2–0.7)	0.5 (0.2–1.0)
Moderate regular or high regular	69/2131	0.5 (0.3–0.7)	0.6 (0.4–0.9)	34/1317	0.5 (0.3–0.8)	0.6 (0.3–1.2)	35/814	0.5 (0.3–0.8)	0.6 (0.3–1.1)

Association in cohort I (risk cohort), presented with odds ratios (ORs) and 95 % confidence intervals (95 % CI)

^a^ Cohort I: Risk cohort including individuals with no neck pain at baseline

^b^Adjusted for age, alcohol consumption, immigration status, work above shoulder level, work under knee level and computer work

^c^Adjusted for age, smoking, immigrant status, psychological distress, working with arms above shoulder level, work underneath knee level and computer work

^d^Adjusted for age, alcohol consumption, smoking, individual disposable income, immigrant status, work underneath knee level and computer work

In the analysis of both sexes combined, the results showed lower odds of getting LDNP in all active categories compared to the sedentary. Being physically active was associated with 30–40 % reduction of the odds of getting LDNP. The sex stratified results had lower precision and there were no clear differences between men and women (Table 3).

### Cohort II - Prognostic cohort

The baseline characteristics of the study sample (*n* = 6 820) are shown in Table 1. The mean age of the participants was 42 (SD 11) years and the study sample consisted of 42 % men.

#### Work related physical activity

Table 4 displays the crude and adjusted ORs of association between work related physical activity and LDNP among participants with occasional neck pain.

**Table 4:** Association between work related physical activity and long duration troublesome neck pain in cohort II^a^

	Both sexes OR (95 % CI)			Men OR (95 % CI)			Women OR (95 % CI)		
Work related physical activity	Cases/non-cases	Crude	Adjusted^b^	Cases/non-cases	Crude	Adjusted^c^	Cases/non-cases	Crude	Adjusted^d^
Sedentary (ref)	293/2 628	1	1	106/1 127	1	1	187/1 501	1	1
Active but not heavy	224/1 738	1.2 (1.0–1.4)	1.1 (0.9–1.3)	83/694	1.3 (0.9–1.7)	1.1 (0.8–1.6)	141/1 044	1.1 (0.9–1.4)	1.0 (0.8–1.3)
Active and moderately heavy or Heavy	235/1 638	1.3 (1.1–1.5)	1.0 (0.8–1.3)	104/749	1.5 (1.1–2.0)	1.0 (0.6–1.5)	131/889	1.2 (0.9–1.5)	1.0 (0.8–1.3)

Association in cohort II (prognostic cohort), presented with odds ratios (ORs) and 95 % confidence intervals (95 % CI)

^a^Cohort II: Prognostic cohort including individuals with occasional neck pain at baseline

^b^Adjusted for age, individual disposable income, work above shoulder level

^c^Adjusted for age, smoking, individual disposable income, work under knee level and work over shoulder level

^d^Adjusted for age, immigrant status and work over shoulder level

The adjusted odds of getting LDNP were close to one in all active categories compared to the sedentary in the analysis of men and women combined, a result that was seen also for each sex respectively.

#### Leisure physical activity

Table 5 displays the crude and adjusted odds for developing LDNP associated with leisure physical activity among participants with occasional neck pain.

**Table 5:** Association between leisure physical activity and long duration troublesome neck pain in cohort II^a^

	All OR (95 % CI)			Men OR (95 % CI)			Women OR (95 % CI)		
Leisure physical activity	Cases/non-cases	Crude	Adjusted^b^	Cases/non-cases	Crude	Adjusted^c^	Cases/non-cases	Crude	Adjusted^d^
Sedentary (ref)	128/888	1	1	57/492	1	1	71/459	1	1
Moderate	357/2 690	0.9 (0.7–1.1)	1.0 (0.8–1.3)	147/1 061	1.0 (0.8–1.4)	1.2 (0.8–1.7)	210/1 629	0.8 (0.6–1.1)	0.9 (0.6–1.2)
Moderate, regular or High regular	268/2 421	0.8 (0.6–1.0)	0.9 (0.7–1.1)	92/1 075	0.6 (0.5–0.9)	0.8 (0.5–1.1)	176/1 346	0.8 (0.6–1.1)	0.9 (0.7–1.2)

Association in cohort II (prognostic cohort), presented with odds ratios (ORs) and 95 % confidence intervals (95 % CI)

^a^Cohort II: Prognostic cohort including individuals with occasional neck pain at baseline

^b^Adjusted for age and immigration status

^c^Adjusted for age, immigrant status, work over shoulder level and computer work

^d^Adjusted for age, smoking and immigrant status

The adjusted odds of getting LDNP were close to one in all active categories compared to the sedentary in the analysis of men and women combined, and in sex stratified analyses.

## Discussion

The adjusted results of this study suggest that being physically active during leisure time has a protective effect on the risk of developing LDNP. Our results show no clear difference between the sexes regarding this effect. Leisure physical activity did not, however, affect the prognosis of neck pain.

Neither the risk of developing LDNP nor the prognosis of neck pain was affected by work related physical activity in our study.

Physical activity has been investigated previously as a potentially protective factor for the risk of developing neck pain, but a systematic review concludes that the results vary [4]. A recent prospective study by Nielsen et al. found that leisure physical activity had a slightly protective effect on chronic neck pain, [21] which is in accordance with our results. They also concluded that leisure physical activity was beneficial even at a relatively low level, compared to inactivity. Contradictory results were found in another prospective study by Kaaria et al. who in an exploratory model investigated the association between leisure physical activity and chronic neck pain among employees. They found that there was no crude association [22]. These inconclusive findings may be due to different measurement of the exposure or different contexts of measuring the exposure.

There are also a limited number of previous studies on the prognosis for neck pain and associations with physical activity. Our results support earlier findings of no association between the exposures and the prognosis for neck pain, although these studies focused on improvement of neck pain while our study focused on worsening of neck pain [23, 24].

The individuals in the prognostic cohort did not have frequent neck pain at baseline. This was operationalized in order to have a distinguished difference between the baseline neck pain and the outcome, LDNP. Since we found an association between the exposure and outcome in the risk cohort but not in the prognostic cohort, we believe that we have found a clinically meaningful division between the two cohorts.

Our results did not support our hypothesis that physical activity would be beneficial for both risk and prognosis of neck pain. Also contradicting our hypothesis, the results suggest that work related physical activity and leisure physical activity affect the risk of neck pain differently. One potential explanation for this is that there may be mediating factors contributing to the positive effect of leisure physical activity, compared to work related physical activity. If physical activity is self-chosen, as it often is during leisure time, it may lead to a more positive, stress relieving effect, and thus counteract neck pain. At the work place the physical activity is often part of the job description and not self-chosen, and may therefore not have the same positive effect. Another possible explanation is that persons choosing to be physically active during leisure time are more health conscious overall, compared to the general population, which could potentially affect neck pain. Results from a recent study suggest that a healthy lifestyle behavior is associated with a decreased risk of low back pain [25].

The results of our study suggest that physical activity during leisure time prevents long-duration troublesome neck pain if you are free from neck pain, but not if you already are mildly affected by neck pain. A clinical implication derived from our results is that physical activity may be important for primary prevention, but when the neck pain is already established other strategies may be needed to hinder it from progressing to more severe neck pain.

This prospective population-based study contains information on a wide variety of domains, which is a major strength. The rich data gave an opportunity for an extensive analysis of confounding which vouches for high internal validity, although we cannot totally rule out unmeasured or residual confounding.

This study also has limitations that need to be addressed. Leisure physical activity and work related physical activity are exposures that can vary over time, and as the time span between baseline and follow-up is five years, a change in level of physical activity during this time could lead to biased estimates. If a possible misclassification would be non-differential it would lead to an underestimation of the true effect, at least when comparing extremes. Non-differential misclassification of an exposure means that the exposure is incorrectly classified to the same extent among those who become cases and those who do not become cases. Speaking against that this possible bias would have a significant effect on our results, is a study from Hamer et al. which found that a higher level of physical activity in middle age is associated with a higher level of physical activity later in life [26]. This indicates that physically active behavior is relatively persistent over time. Our measurement method for physical activity has been reported to be useful for categorizing adults into different levels of PA, based on the physical activity in the different groups as measured by accelerometer [27]. Considering work related physical activity, it is hard to say whether the exposure is likely to change or not. If you have neck pain at baseline it is possible that you try to change your work tasks if you believe that they are contributing to the pain. Our general perception however, is that it is not often possible for individuals to change their work tasks due to neck problems. In addition, it is not given in what direction individuals would want to change their activity, to be more or less active, depending on their beliefs about what would reduce the neck problems.

Despite the fact that the exposure measurements in this study are rough we believe that we, through the questions, are able to distinguish between people that are sedentary or more active and if they have higher or lower work related physical activity in a decent manner. According to previous studies self-administered questionnaires are able to classify participants to sedentary or standing work and low or high workload in acceptable accordance with interview [28] and observational [29] data. Furthermore Sabbath et al. concluded that a single item question was a fairly good indicator of physical load at work compared to a 38-item questionnaire [30]. The authors found the single item work related physical activity question to be sensitive enough to detect increased levels of work related physical activity within a certain task, for example lifting or pulling, and also to detect overall increases in total work related physical activity.

The outcome LDNP may also be sensitive to measurement error (reporting bias). This is a possible source of bias. The size of such a bias and its influence on the results is, if any, likely to be non- differential (not differ across the exposure groups). Thus it is possible that such measurement error is diluting the association between the exposure and the outcome. We do believe however, that since the outcome is defined as having had considerably bothersome neck pain for a period of at least three consecutive months, most participants would recall such an episode during a period of five years. Therefore the dilutive effect would refrain from being a major issue.

In both the sub cohort with no neck pain at baseline, as well as the sub cohort with occasional neck pain at baseline, the loss to follow-up was 21 %. For selection bias to occur, both the prevalence of the exposure and the outcome has to differ between the drop-outs and those successfully followed, [30] which we find unlikely regarding the exposure leisure physical activity. Thus since we had almost an 80 % response rate we do not think that the validity of the study is affected to a notable extent. It may be more likely that it would differ regarding work related physical activity, with a higher proportion of LDNP cases reporting pain at follow-up since neck pain has a bigger impact on daily activities if you have a physically active work.

## Conclusion

In conclusion our results suggest that being physically active during leisure time reduces the risk of developing LDNP if you initially are free from neck problems. However leisure physical activity did not affect the prognosis for neck pain once it is already established.

Work related physical activity affects neither the risk nor the prognosis of neck pain in our study.

The results indicate that leisure physical activity is a factor to consider in primary prevention for neck pain, which is a result in line with public health recommendations for primary prevention for other public health diseases.

### Ethics and consent for publication

This cohort study was approved by the Ethical review board in Stockholm, Sweden, Diary number 2007/545-31 and 2009/457-31. Informed consent was received from all study participants, including consent for publication of the results.

### Consent for publication

Not applicable.

### Availability of data and materials

The dataset supporting the conclusions of this article is stored at the Institute of Environmental Medicine, Karolinska Institutet, Stockholm, Sweden. Data will not be shared or available in an open access repository because the authors have not finished the data analysis yet. If anyone is interested in exploring specific issue, please contact ES.

## Abbreviations

CI: confidence interval
GHQ12: general health questionnaire
JCQ: job content questionnaire
LDNP: long duration troublesome neck pain
OR: odds ratio
SPHC: Stockholm Public Health Cohort
